# 

**Published:** 1900-10-15

**Authors:** 


### ANNOUNCEMENTS.

### MEETING OF THE OHIO STATE BOARD OF DENTAL EXAMINERS.

The next meeting of this Board will be held in Columbus, Ohio, beginning the last Tuesday in November, 1900. All those desiring to take the examination will receive full instructions and application blanks by applying to the Secretary, DR. L. P. BETHEL, Kent, Ohio.

### THE OHIO STATE DENTAL SOCIETY.

The thirty-fifth annual meeting will be held at Columbus, Ohio, December 4, 5 and 6, 1900, at the Great Southern Hotel. S. D. RUGGLES, *Sec'y.*

